# Lin28a protects against cardiac ischaemia/reperfusion injury in diabetic mice through the insulin-PI3K-mTOR pathway

**DOI:** 10.1111/jcmm.12369

**Published:** 2015-02-16

**Authors:** Mingming Zhang, Dongdong Sun, Shuang Li, Xietian Pan, Xiaotian Zhang, Di Zhu, Congye Li, Rongqing Zhang, Erhe Gao, Haichang Wang

**Affiliations:** a Department of Cardiology, Xijing Hospital, Fourth Military Medical UniversityXi'an, China; b Center for Translational Medicine, Temple University School of MedicinePhiladelphia, PA, USA

**Keywords:** diabetes, ischaemia/reperfusion injury, Lin28a

## Abstract

The insulin-PI3K-mTOR pathway exhibits a variety of cardiovascular activities including protection against I/R injury. Lin28a enhanced glucose uptake and insulin-sensitivity *via* insulin-PI3K-mTOR signalling pathway. However, the role of lin28a on experimental cardiac I/R injury in diabetic mice are not well understood. Diabetic mice underwent 30 min. of ischaemia followed by 3 hrs of reperfusion. Animals were randomized to be treated with lentivirus carrying lin28a siRNA (siLin28a) or lin28a cDNA (Lin28a) 72 hrs before coronary artery ligation. Myocardial infarct size (IS), cardiac function, cardiomyocyte apoptosis and mitochondria morphology in diabetic mice who underwent cardiac I/R injury were compared between groups. The target proteins of lin28a were examined by western blot analysis. Lin28a overexpression significantly reduced myocardial IS, improved LV ejection fraction (LVEF), decreased myocardial apoptotic index and alleviated mitochondria cristae destruction in diabetic mice underwent cardiac I/R injury. Lin28a knockdown exacerbated cardiac I/R injury as demonstrated by increased IS, decreased LVEF, increased apoptotic index and aggravated mitochondria cristae destruction. Interestingly, pre-treatment with rapamycin abolished the beneficial effects of lin28a overexpression. Lin28a overexpression increased, while Lin28a knockdown decreased the expression of IGF1R, p-Akt, p-mTOR and p-p70s6k after cardiac I/R injury in diabetic mice. Rapamycin pre-treatment abolished the effects of increased p-mTOR and p-p70s6k expression exerted by lin28a overexpression. This study indicates that lin28a overexpression reduces IS, improves cardiac function, decreases cardiomyocyte apoptosis index and alleviates cardiomyocyte mitochondria impairment after cardiac I/R injury in diabetic mice. The mechanism responsible for the effects of lin28a is associated with the insulin-PI3K-mTOR dependent pathway.

## Introduction

The rising prevalence and incidence of diabetes mellitus (DM) have been reported in response to increasing rates of overweight, obesity and more sedentary lifestyle 1–3. To our knowledge, the risk of coronary heart disease (CHD) is about three to fourfold higher in diabetic patients, while diabetes is associated with two to four times increased risk of CHD mortality compared with patients without diabetes 4,5. These findings emphasize the need for increasing efforts to prevent DM and to aggressively treat and control cardiovascular disease (CVD) risk factors among diabetic patients 6.

MicroRNAs and RNA-binding proteins are emerging as pivotal modulators of mammalian cardiovascular development and disease 7–12. Lin28, a developmentally regulated RNA-binding protein, selectively blocks the production of let-7 miRNAs 8. When overexpressed in mice, Lin28a promoted an insulin-sensitized state that resisted high-fat diet-induced diabetes. On the contrary, muscle-specific loss of lin28a and overexpression of let-7a resulted in insulin resistance and impaired glucose tolerance 13. These data establish the Lin28/let-7 pathway as a central regulator of mammalian glucose metabolism.

The insulin-PI3K-mTOR pathway exhibits a variety of cardiovascular activities including protection against I/R injury 14. Zhu *et al*. reported that the mTOR inhibitor rapamycin abrogated the enhanced glucose uptake and insulin-sensitivity conferred by lin28a *in vitro* and *in vivo*, indicating that lin28a enhanced glucose uptake and insulin-sensitivity *via* insulin-PI3K-mTOR signalling pathway 13,15,16. However, the role of lin28a on experimental cardiac I/R injury in diabetic mice is not well understood. The aims of the present study were to (*i*) determine whether lin28a protects diabetic mice from cardiac I/R injury and (*ii*) identify whether the underlying mechanisms is associated with the insulin-PI3K-mTOR dependent pathway.

## Methods

### Animals and surgical protocols

Male C57BL/6 mice received humane care in adherence with the National Institutes of Health Guidelines on the Use of Laboratory Animals and were approved by the Fourth Military Medical University Committee on Animal Care. Diabetic mice were induced in adult male C57BL/6 mice (6–8 weeks), weight 20–25 g by intraperitoneal (i.p.) injections of Streptozocin (STZ; 50 mg/kg, STZ was dissolved in 0.1 M citrate buffer, pH 4.5) for 5 days and high-fat diets for 2 months. A one-drop blood sample was obtained at 8 weeks from all mice through the tip of the tail for the determination of blood glucose concentration by using a reflectance meter (Accu-Chek, Roche Diagnostics GmbH, Mannheim, Germany). Animals with glucose levels not less than 16.6 mmol/l were classified as diabetes. Mice were randomly divided into the following groups with *n* = 20 each: (*i*) Non-DM; (*ii*) DM + sham (Sham); (*iii*) DM + I/R (I/R); (*iv*) DM + siControl + I/R (I/R + siControl); (*v*) DM + Lin28a siRNA + I/R (I/R +siLin28a); (*vi*) DM + Control vector+ I/R (I/R + Control vector); (*vii*) DM + Lin28a Overexpression + I/R (I/R + Lin28a) and (*viii*) DM + Lin28a Overexpression + Rapamycin + I/R (I/R + Lin28a + RAP).

Ten weeks after high-fat diets was given, diabetic mice were anesthetized with 2% isoflurane, and the heart was exposed *via* left fifth intercostal space thoracotomy. Lentivirus (30 μl, 1 × 10^9^ TU/ml) was delivered *via* three separate intramyocardial injections, temporarily blanching the LV free wall. Hearts were subjected to I/R injury 72 hrs after lentivirus injection 17. Rapamycin (a specific mTOR inhibitor, 5 mg/kg) was injected *via* the tail vein 10 min. before cardiac I/R in the I/R + Lin28a + RAP group.

### Cardiac I/R injury model construction

Cardiac I/R injury model was constructed as previously described 18. A left thoracic incision was used to open the chest. A 6–0 silk suture slipknot was placed at the proximal one-third of the left anterior descending artery. After 30 min. of ischaemia, the slipknot was released, and the myocardium was reperfused for 3 hrs. Sham group underwent the same surgical protocols except that the suture placed under the left coronary artery was not tied.

### Measurement of myocardial infarct size

After 3 hrs reperfusion, the ligature around the coronary artery was retied, and 1 ml of 2% Evans Blue dye was injected into the side arm of the aortic cannula. When the dye was well- distributed, the heart was quickly excised, frozen at −80°C and sliced transversally into 1 mm thick sections. The slices were incubated in 1%2,3,5-triphenyltetrazoliumchloride (TTC; Sigma-Aldrich, St Louis, MO, USA) for 30 min. at 37°C as previously described 18. Blue areas which were stained by Evans Blue indicated area not at risk (ANAR). TTC stained areas which were red parts in the heart represented ischaemic but viable tissue. Staining negative areas indicated infracted myocardium. Areas of infarct size (IS) and area at risk (AAR) were measured digitally by using IMAGE PRO PLUS software (Media Cybernetics, Bethesda, MD, USA). IS and AAR were expressed as percentages of the LV area (IS/LV and AAR/LV respectively).

### Determination of myocardial apoptosis

Myocardial apoptosis was determined by terminal deoxyribonucleotidyl transferase-mediated dUTP-biotin nick end labelling (TUNEL) staining as previously described 19. TUNEL staining was performed with fluorescein-dUTP (In Situ Cell Death Detection Kit; Roche Diagnostics) for apoptotic cell nuclei and 4,6-diamidino-2-phenylindole (DAPI; Sigma-Aldrich) stained all cell nuclei. AI is the number of TUNEL-positive myocytes divided by the total number of myocytes stained with DAPI from a total of 40 fields per heart (*n* = 5). Cleaved Caspase-3, Caspase-3, Bcl-2, Bax were detected by Western Blot evaluation. All of these assays were performed in a blinded manner.

### Determination of cardiac function

Echocardiography performed at 24 hrs after reperfusion as previously described 18. Sedated mice (2% isoflurane) were studied on an echocardiography system (Sequoia Acuson, 15-MHz linear transducer; Siemens, Erlangen, Germany). Cardiac dimensions and function were assessed by M-mode echocardiography. LV end-diastolic diameter and LV end-systolic diameter were measured on the parasternal LV long axis view. All measurements represent the mean of 5 consecutive cardiac cycles. LV end-systolic volume (LVESV), LV end-diastolic volume (LVEDV) and LV ejection fraction (LVEF) were calculated by the use of computer algorithms. All of these measurements were performed in a blinded manner. The LV pressure was measured *via* a Millar Mikro-tip catheter transducer that was inserted into the LV cavity through the left carotid artery. The LV systolic pressure, LV end-diastolic pressure, first derivative of the LV pressure (±LV dp/dt max) and heart rate were obtained by use of computer algorithms and an interactive videographics programme (Po-Ne-Mah Physiology Platform P3 Plus; Gould Instrument Systems, Valley View, OH, USA).

### Determination of myocardium IL-6, TNF-α and MPO activity

The concentrations of interleukin-6 (IL-6) and tumour necrosis factor-alpha (TNF-α) were measured by ELISA kits according to the manufacturer's instructions. Values were expressed as pictograms per milligram of total protein. Following the 3 hrs reperfusion period, tissue samples were taken from the AAR zones for myeloperoxidase (MPO) activity analysis. The activity of MPO was measured spectrophotometrically at 460 nm and expressed as units per 100 mg of tissue.

### Quantitative real-time PCR (qRT-PCR) analysis

Total RNA was extracted by using TRIZOL reagent (Invitrogen, Carlsbad, CA, USA) according to the manufacturer's protocol. The first strand cDNA was generated from total RNA with reverse transcriptase (TAKARA, Shiga, Japan) and used as the template for qRT-PCR analysis. GAPDH cDNA was used as an internal control to normalize variances. Primers used were as follows: lin28a, 50-GAGGCAGTGGAGTTCACCTTTA-30 (forward) and 50-TCCTTGGCATGGTGGTCTA-30 (reverse); GAPDH, 50-GGCACAGTCAAGGCTGAGAATG-30 (forward) and 50-ATGGTGGTGAAGACGCCAGTA-30 (reverse); let7a, 50-CGGTGAGGTAGTAGGTTGTATAGTT-30 (forward). PCR was performed in a GeneAmp PCR system 2400 Thermal Cycler (Perkin-Elmer, Norwalk CT, USA). PCR conditions were 30 sec. at 94°C, 30 sec. at 58°C and 30 sec. at 72°C (30 cycles). The PCR products were detected by real-time PCR detection kit (RR716; TAKARA) in ABI 7500 sequence detection system.

### Western blot evaluation

Total proteins from tissues were separated by SDS-PAGE, blotted and probed with anti-Akt (Cell Signaling, Danvers, MA, USA), anti-phospho-Akt (ser473; Cell Signaling), anti-β-actin antibody (Santa Cruz, CA, USA), anti-Lin28a (Abcam, Cambridge, MA, USA), anti-p70S6k (Cell Signaling), anti-p-p70S6k (Thr389, Cell Signaling), anti-Cleaved Caspase-3(Sigma-Aldrich), anti-Caspase-3 (Sigma-Aldrich), anti-Bcl-2 (Sigma-Aldrich), anti-Bax (Sigma-Aldrich). The Bradford assay (Bio-Rad Laboratories, Hercules, CA, USA) was used to quantify protein concentrations. The blots were visualized with a chemiluminescence system (Amersham Bioscience, Buchinghamshire, UK). The signals were quantified by densitometry.

### Lentiviral-vectored Lin28a siRNA and Lin28a cDNA

Lentivirus carrying Lin28a siRNA or Lin28a cDNA were purchased from Gene-Pharma Company (Shanghai, China). The RNAi sequence targeting mouse lin28a is 50-GCAGTGGAGTTCACCTTTAAG-30.

### Statistical analysis

All values and figures were expressed as mean ± SD. Comparison between groups was subjected to ANOVA followed by Bonferroni correction for post hoc *t*-test. Two-sided tests have been used and *P* < 0.05 were considered statistically significant. SPSS software package version 14.0 (SPSS, Chicago, IL, USA) was performed for data analysis.

## Results

### Basic parameters

There was no major difference between groups in terms of heart rate, blood glucose, body mass before the cardiac I/R injury except that blood glucose and body mass were significantly lower in the non-diabetic mice compared with the diabetic mice (Table S1).

### Lin28a overexpression inhibits, while lin28a knockdown promotes let7a expression after cardiac I/R injury in mice with diabetes

**Figure 1:**
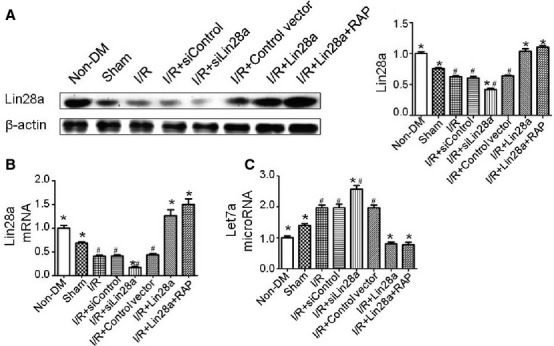
Validation of lin28a overexpression and lin28a knockdown. Lin28a expression levels with representative gel blots of lin28a and β-actin (loading control) were shown (A); Lin28a mRNA expression as evaluated by real-time PCR analysis (B); Let7a (microRNA) expression as evaluated by real-time PCR analysis (C). Columns and bars represent mean ± SD. *n* = 5–6 per group. *P < 0.05 *versus* I/R, ^#^*P* < 0.05 *versus* I/R + Lin28a.

Lin28a overexpression and lin28a knockdown model were successfully constructed and confirmed by western blot and real-time PCR analysis (Fig.1A and B). Lin28a overexpression inhibited, while lin28a siRNA administration promoted let7a expression as demonstrated by real-time PCR analysis (Fig.1C). CTs values were presented in Table S2.

### Lin28a overexpression decreases, while lin28a siRNA administration increases IS after cardiac I/R injury in diabetic mice

**Figure 2:**
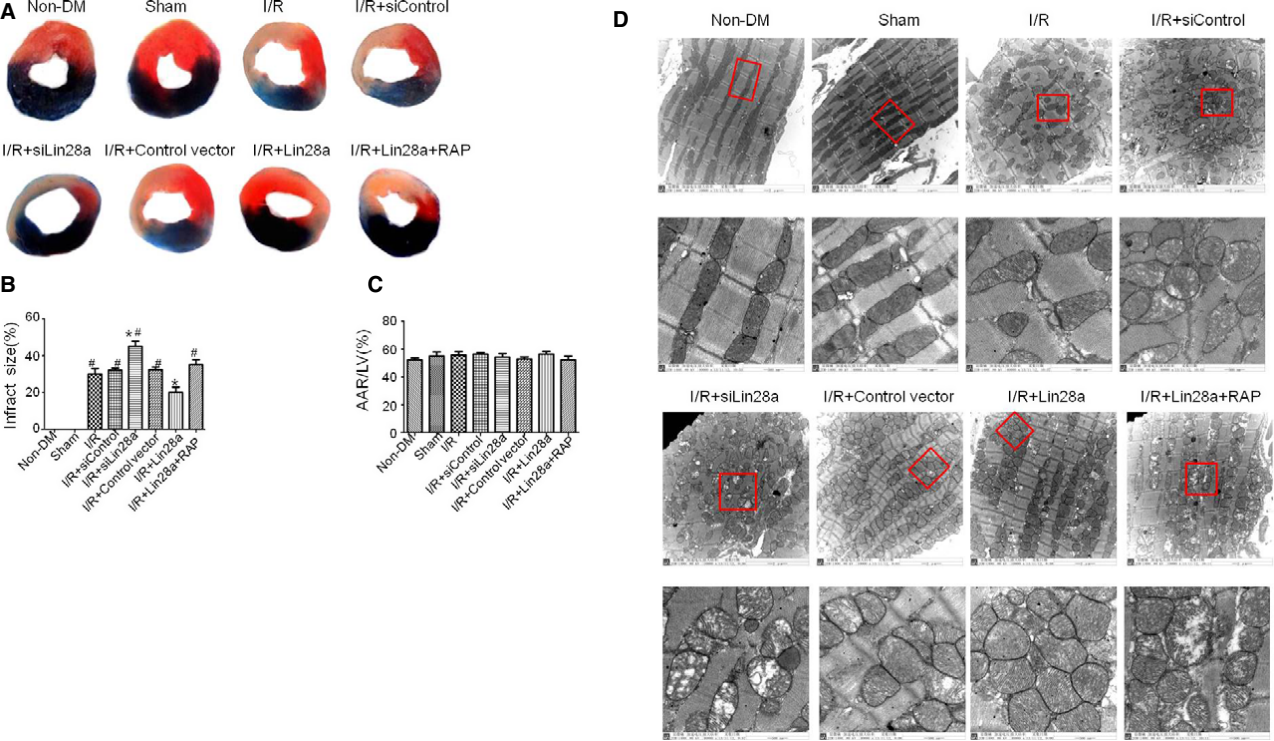
Lin28a overexpression decreases infarct size (IS) after cardiac ischaemia/reperfusion (I/R) injury in mice with diabetes. Representative illustrations of IS as stained by Evans Blue and 2,3,5 -triphenyltetrazoliumchloride (TTC; A). IS measured at 3 hrs after I/R injury (B). No significant difference in risk area are found between groups (C). Cardiomyocytes ultrastructure changes as evaluated by transmission electron microscopy (D). Columns and bars represent mean ± SD. *n* = 6–7 per group. Blue areas which were stained by Evans Blue indicated area not at risk (ANAR). TTC stained areas which were red parts in the heart represented ischaemic but viable tissue. Staining negative areas indicated infracted myocardium. Area at risk (AAR) represented ischaemic but viable tissue and infracted myocardium. *P < 0.05 *versus* I/R, ^#^*P* < 0.05 *versus* I/R + Lin28a.

Representative illustrations of IS as stained by Evans Blue and TTC were shown in Figure2A. Lin28a overexpression (I/R + Lin28a) decreased IS (0.212 ± 0.028 *versus* 0.317 ± 0.019, *P* < 0.05), while lin28a siRNA administration (I/R + siLin28a) increased IS at 3 hrs (0.415 ± 0.018 *versus* 0.317 ± 0.019, *P* < 0.05) after cardiac I/R injury in diabetic mice compared with the I/R group. Rapamycin pre-treatment abolished the beneficial effects of lin28a overexpression on IS (I/R + Lin28a + RAP: 0.352 ± 0.016; I/R + Lin28a: 0.212 ± 0.028, *P* < 0.05; Fig.2B). No significant difference in risk area was found between the eight groups (Fig.2C).

### Lin28a overexpression alleviates cardiomyocytes ultrastructure impairment induced by cardiac I/R injury in diabetic mice

Electron microscope showed that the number of cardiomyocytes mitochondria was increased in the diabetic group. Cardiomyocytes mitochondria were found greater in size with the destruction of cristae after cardiac I/R (30 min./3 hrs) injury in diabetic mice. Lin28a overexpression alleviated mitochondria ultrastructure impairment as demonstrated by intact membrane, normalized cristae density and architecture. On the contrary, Lin28a siRNA administration aggravated mitochondria ultrastructure impairment as compared with the I/R group. The cristae and matrix were cleared out resulting in vacuoles in most of the swelling mitochondria. Cristae disorientation and breakage were also found in most of the mitochondria. As expected, rapamycin pre-treatment abolished the effects of Lin28a protecting against cardiomyocytes mitochondria ultrastructure impairment (Fig.2D).

### Lin28a preserves LV function after cardiac I/R injury in diabetic mice

**Figure 3:**
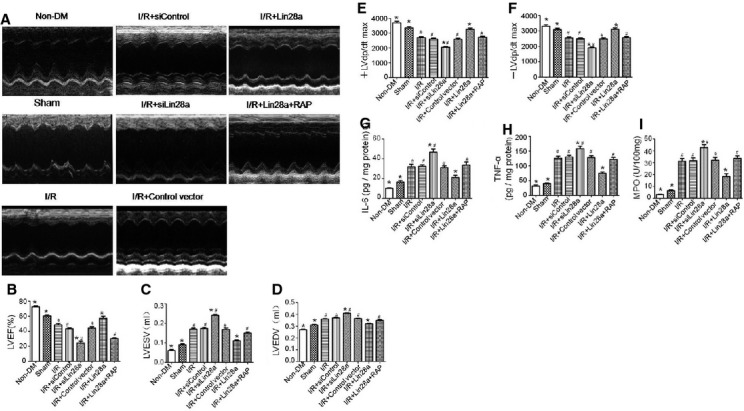
Echocardiographic properties and IL-6, TNF-α and MPO expressions were analysed. Representative echocardiographic images were shown (A); LV ejection fraction (LVEF; B); LV end-diastolic volume (LVEDV; C); LV end-systolic volume (LVESV; D); first derivative of the LV pressure (±LV dp/dt max; E and F); IL-6, TNF-α and MPO expression (G–I). The columns and error bars represent means and SD. *n* = 12–13 mice per group. *P < 0.05 *versus* I/R, ^#^*P* < 0.05 *versus* I/R + Lin28a.

LV ejection fraction, ESV, EDV were evaluated by echocardiography. Diabetic mice in the I/R + Lin28a group had significantly smaller decreases in LVEF (0.494 ± 0.048 *versus* 0.406 ± 0.052, *P* < 0.05) compared with I/R group. LVEF was also significantly higher in the I/R+ Lin28a group compared with the I/R+ Lin28a +RAP group (0.494 ± 0.048 *versus* 0.393 ± 0.055, *P* < 0.05; Fig.3A and B). Lin28a overexpression significantly inhibited the increase of LVESV and LVEDV compared with the I/R group (LVESV: 0.12 ± 0.04 *versus* 0.17 ± 0.02 ml, *P* < 0.05; LVEDV: 0.33 ± 0.04 *versus* 0.36 ± 0.05 ml, *P* < 0.05). Rapamicin pre-treatment abolished the beneficial effects of Lin28a overexpression on LVESV (I/R + Lin28a + RAP: 0.16 ± 0.03; I/R + Lin28a: 0.12 ± 0.04 ml, *P* < 0.05) and LVEDV (I/R + Lin28a + RAP: 0.35 ± 0.05; I/R + Lin28a: 0.33 ± 0.04 ml, *P* < 0.05). Lin28a siRNA administration increased LVESV (0.23 ± 0.03 *versus* 0.17 ± 0.02 ml, *P* < 0.05) and LVEDV (0.41 ± 0.04 *versus* 0.36 ± 0.05 ml, *P* < 0.05) as compared with the I/R group (Fig.3C and D).

Hemodynamic measurements were performed 3 hrs after cardiac I/R injury in diabetic mice. The ±LV dp/dt max was decreased after cardiac I/R injury in diabetic mice (+LV dp/dt max: 2689.9 ± 115.8 *versus* 3359.3 ± 140.3 mmHg/sec., *P* < 0.05; −LV dp/dt max: 2538.5 ± 131.4 *versus* 3086.7 ± 165.1 mmHg/sec., *P* < 0.05). Lin28a overexpression significantly enhanced +LV dp/dt max (3259.4 ± 161.0 *versus* 2689.9 ± 215.8 mmHg/sec., *P* < 0.05) and −LV dp/dt max (3089.9 ± 155.8 *versus* 2538.5 ± 131.4 mmHg/sec., *P* < 0.05) compared with the I/R group. The ±LV dP/dt max was further decreased in the I/R+ siLin28a group (+LV dp/dt max: 2052.4 ± 125.7 *versus* 2689.9 ± 215.8 mmHg/sec., *P* < 0.05; −LV dp/dt max: 1923.7 ± 149.7 *versus* 2538.5 ± 131.4 mmHg/sec., *P* < 0.05) compared with the I/R group. Rapamicin pre-treatment abolished the beneficial effects of lin28a overexpression on ±LV dP/dt max (+LV dp/dt max: 2720.4 ± 129.6 *versus* 3278.4 ± 165.0 mmHg/sec., *P* < 0.05; −LV dp/dt max: 2570.5 ± 153.4 *versus* 3089.9 ± 115.8 mmHg/sec., *P* < 0.05) compared with the I/R+ Lin28a group (Fig.3E and F).

### Lin28a reduces cytokine levels and alleviates leucocyte infiltration after cardiac I/R injury in diabetic mice

The levels of LV IL-6, TNF-α and MPO were measured (Fig.3G–I). Cardiac I/R injury resulted in a noticeable increase in IL-6 (31.7 ± 4.6 *versus* 16.5 ± 2.7 pg/mg protein, *P* < 0.05), TNF-α (126.6 ± 12.3 *versus* 40.5 ± 4.7 pg/mg protein, *P* < 0.05) and MPO (31.2 ± 3.0 *versus* 6.5 ± 1.1U/100 mg, *P* < 0.05) expression as compared with the Sham group. Lin28a overexpression reduced the levels of IL-6 (21.0 ± 3.9 *versus* 31.7 ± 4.6 pg/mg protein, *P* < 0.05), TNF-α (75.2 ± 8.3 *versus* 126.6 ± 12.3 pg/mg protein, *P* < 0.05) and MPO (18.3 ± 4.2 *versus* 31.2 ± 4.1 U/100 mg, *P* < 0.05) compared with the I/R group. The effect of lin28a on IL-6, TNF-α and MPO production was abolished by rapamycin pre-treatment (IL-6 33.5 ± 5.6 *versus* 21.0 ± 3.9 pg/mg protein, *P* < 0.05; TNF-α 122.8 ± 12.6 *versus* 75.2 ± 8.3 pg/mg protein, *P* < 0.05; MPO 33.7 ± 3.7 *versus* 18.3 ± 4.2 U/100 mg, *P* < 0.05). Lin28a siRNA administration increased in IL-6 (46.7 ± 5.3 *versus* 31.7 ± 4.6 pg/mg protein, *P* < 0.05), TNF-α (158.6 ± 14.3 *versus* 126.6 ± 12.3 pg/mg protein, *P* < 0.05) and MPO (42.7 ± 4.2 *versus* 31.2 ± 4.1 U/100 mg, *P* < 0.05) expression compared with the I/R group.

### Lin28a overexpression inhibits cardiomyocytes apoptosis after cardiac I/R injury in diabetic mice

**Figure 4:**
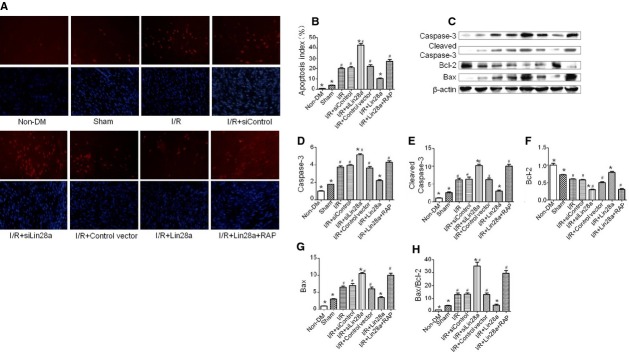
Anti-apoptotic effect of lin28a overexpression on cardiomyocytes after cardiac I/R injury in diabetic mice. Terminal deoxyribonucleotidyl transferase-mediated dUTP-biotin nick end labelling (TUNEL) -positive myocytes were indicated in red (A). Quantitative analysis of apoptotic cardiomyocytes isolated from ischaemic cardiac tissue in diabetic mice (B). Protein expression with representative gel blots of Caspase-3, Cleaved Caspase-3, Bcl-2, Bax and β-actin (loading control; C–H). The columns and error bars represent means and SD. *n* = 6–7 mice per group. *P < 0.05 *versus* I/R, ^#^*P* < 0.05 *versus* I/R + Lin28a.

Compared with the I/R group, representative photomicrograph showed that TUNEL-positive cardiomyocytes were less observed in lin28a overexpression group (0.125 ± 0.026 *versus* 0.211 ± 0.013, *P* < 0.05). Quantitative analyses demonstrated that the percent of TUNEL-positive cardiomyocytes was significantly higher in the I/R + siLin28a group (0.414 ± 0.017 *versus* 0.211 ± 0.013, *P* < 0.05) compared with the I/R group. Rapamycin pre-treatment abolished the beneficial effects of lin28a overexpression on cardiomyocytes apoptosis (I/R + Lin28a + RAP: 0.204 ± 0.025, I/R + Lin28a: 0.125 ± 0.026, *P* < 0.05) compared with the I/R + Lin28a group (Fig.4A and B). Cleaved Caspase-3, Caspase-3 and Bax protein levels evaluated by western blot were down-regulated by lin28a overexpression and up-regulated by lin28a siRNA administration. The pro- to anti-apoptotic protein (Bax/Bcl-2) ratio were significantly decreased in the I/R+ Lin28a group, while increased in the I/R+ siLin28a group. Furthermore, I/R + Lin28a +RAP administration increased Bax/Bcl-2 ratio, Cleaved Caspase-3, Caspase-3, Bax protein levels and decreased Bcl-2 protein level in ischaemic cardiac tissue compared with the I/R+ Lin28a group (Fig.4C–H).

### Lin28a overexpression increases IGF1R, p-Akt, p-mTOR, p-p70s6k expression in myocardium exposed to I/R injury in diabetic mice

**Figure 5:**
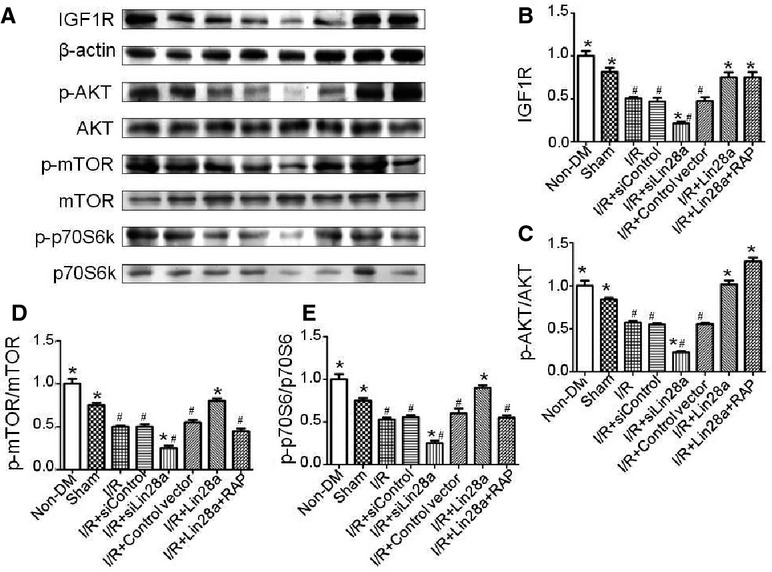
Effect of lin28a overexpression or knockdown on insulin-PI3K-mTOR signalling pathway activation. Hearts were subjected to *in vivo* ischaemia (30 min.) followed by reperfusion (3 hrs). Representative gel bolts depicting respective protein expression by using specific antibodies (A); IGF1R (B); phosphorylated AKT (p-AKT; C); phosphorylated mTOR (p-mTOR; D); phosphorylated p70s6k (p-p70s6k; E). The columns and error bars represent means and SD. *n* = 6–7 mice per group. *P < 0.05 *versus* I/R, ^#^*P* < 0.05 *versus* I/R + Lin28a.

After 3 hrs of reperfusion, western blot analysis revealed that lin28a overexpression was associated with a significant increase in IGF1R and phosphorylation of Akt, mTOR and p70s6k protein in ischaemic cardiac tissue that was exposed to I/R injury in diabetic mice. Rapamycin pre-treatment inhibited phosphorylation of mTOR and p70s6k. Lin28a siRNA administration decreased IGF1R, p-Akt, p-mTOR and p-p70s6k expression levels in ischaemic cardiac tissue in diabetic mice (Fig.5).

## Discussion

The increased prevalence of DM is a major concern for cardiologists. DM is widely acknowledged to significantly increase the risk of developing cardiac death 20. Treatment strategies for diabetic patients require both blood glucose control and adverse cardiovascular events control. These findings warrant the significance of aggressive primary prevention against ischaemia/reperfusion (I/R) injury in diabetic patients. In the present study, lin28a limited IS in diabetic mice after I/R injury. Hemodynamic measurements and echocardiography evaluation revealed preserved LV function by lin28a overexpression.

The irreversible mitochondrial dysfunction is a crucial event in cardiomyocyte death following a myocardial I/R injury. After reperfusion, intracellular Ca^2+^ elevation and increased ROS production trigger mPTP opening, leading to the depletion of ATP and loss of mitochondrial integrity 21,22. Alleviating mitochondrial dysfunction may decrease cardiac I/R injury. Meanwhile, modulating mitochondrial function may improve insulin resistance and reduce subsequent cardiac mortality 23,24. In the present study, lin28a overexpression alleviated mitochondria ultrastructure impairment as demonstrated by intact membrane and cristae although still with a greater size after cardiac I/R in diabetic mice. Lin28a protected against cardiac I/R injury in diabetic mice at least in part by alleviating mitochondrial dysfunction.

Previous studies have shown that a large accumulation of neutrophils was observed in both the permanently occluded and reperfused myocardium, suggesting that an inflammatory response may contributes to apoptosis in both settings 25. In this study, we found that lin28a overexpression inhibited, while lin28a siRNA administration enhanced inflammatory responses by modulating inflammatory factors production including IL-6 and TNF-α after cardiac I/R injury in diabetic mice.

Cardiomyocytes apoptosis is one of the major pathogenic mechanisms underlying myocardial I/R injury. Blocking the apoptosis process could prevent the loss of contractile cells, minimize cardiac injury induced by I/R injury 26,27. Thus, we performed TUNEL staining and measured Caspase-3 activity by western blot to explore the underlying mechanism responsible for the cardiac function improvement induced by lin28a overexpression in diabetic mice after cardiac I/R injury. The results indicated that lin28a overexpression inhibited cardiomyocytes apoptosis after I/R injury in diabetic mice as demonstrated by decreased TUNEL-positive cardiomyocytes and decreased caspase-3 expression levels. On the contrary, lin28a siRNA administration increased cardiomyocytes apoptosis, exacerbated cardiac I/R injury in diabetic mice after cardiac I/R injury.

One of the possible mechanisms which has been proposed for the protective effects of lin28a overexpression is blocking let7 production and inhibiting let-7-mediated repression of multiple components of the insulin-PI3K-mTOR pathway, including IGF1R, INSR and IRS2 13,28. The lin28a/let-7a axis regulated glucose metabolism in part through the insulin-PI3K-mTOR pathway and the insulin-PI3K-mTOR pathway was involved in the cardioprotection against myocardial ischaemia/reperfusion injury 13,14. To explore whether the protective effects of lin28a overexpression are associated with insulin-PI3K-mTOR pathway, the specific mTOR inhibitor rapamycin was employed in the present study. Lin28a overexpression inhibited, while lin28a knockdown enhanced let7a expression. Furthermore, lin28a overexpression increased, while lin28a knockdown decreased IGF1R, p-Akt, p-mTOR and p-p70s6k expression after cardiac I/R injury in diabetic mice. Interestingly, pre-treatment with rapamycin abolished the effects of lin28a overexpression as demonstrated by increased IS, MPO activity, caspase-3 expression and increased production of IL-6 and TNF-α, worsened LV function, enhanced cardiomyocytes apoptosis and decreased p- mTOR and p-p70s6k expression. These results suggest that lin28a overexpression induces cardioprotective effects through the activation of insulin-PI3K-mTOR pathway.

In conclusion, the salient finding of the present study is that lin28a overexpression reduces IS and alleviates cardiac dysfunction after cardiac I/R injury in diabetic mice. This is accompanied by decreased cardiac apoptosis and inflammation. The mechanism responsible for the effects of lin28a overexpression is mediated, at least in part, by the insulin-PI3K-mTOR dependent pathway.

## Limitations

Other members of RISK pathway may have participated in the link between Lin28a and its cardiac protection effects. The function of cardiomyocytes is closely related to mitochondria biogenesis and mitochondrial dysfunction is the primary cause of I/R-induced apoptosis of cardiomyocytes. To further demonstrate the efficacy of Lin28a administration on cardiomyocyte mitochondrial biogenesis and function, AMPK related pathway should be measured in future studies. The protection effects of Lin28a may link to the metabolic derangement. Further studies are needed to investigate whether Lin28a activates Akt phosphorylation by different mechanisms in diabetic mice or non-diabetic mice underwent cardiac ischaemia insult.

## Supporting Information

**Figure S1** Representative gel bolts depicting respective protein expression using specific antibodies **(A)**; Lin28a **(B)**; phosphorylated AKT (p-AKT; C). **Figure S2** Representative gel bolts depicting respective protein expression using specific antibodies **(A)**; phosphorylated AKT (p-AKT; B). **Figure S3** Representative gel bolts depicting respective protein expression using specific antibodies **(A)**; IGF1R **(B)**; phosphorylated AKT (p-AKT; **C**). **Table S1** Basic parameters of the mice. **Table S2** CTs Values of Lin28a and Let7a expression.

## References

[b1] Mokdad AH, Bowman BA, Ford ES (2001). The continuing epidemics of obesity and diabetes in the United States. JAMA.

[b2] Stovring H, Andersen M, Beck-Nielsen H (2003). Rising prevalence of diabetes: evidence from a Danish pharmaco-epidemiological database. Lancet.

[b3] Fox CS, Pencina MJ, Meigs JB (2006). Trends in the incidence of type 2 diabetes mellitus from the 1970s to the 1990s: the Framingham Heart Study. Circulation.

[b4] Gu K, Cowie CC, Harris MI (1998). Mortality in adults with and without diabetes in a national cohort of the U.S. population, 1971-1993. Diabetes Care.

[b5] Lee WL, Cheung AM, Cape D (2000). Impact of diabetes on coronary artery disease in women and men: a meta-analysis of prospective studies. Diabetes Care.

[b6] Fox CS, Coady S, Sorlie PD (2007). Increasing cardiovascular disease burden due to diabetes mellitus: the Framingham Heart Study. Circulation.

[b7] Small EM, Olson EN (2011). Pervasive roles of microRNAs in cardiovascular biology. Nature.

[b8] Viswanathan SR, Daley GQ, Gregory RI (2008). Selective blockade of microRNA processing by Lin28. Science.

[b9] Viswanathan SR, Daley GQ (2010). Lin28: a microRNA regulator with a macro role. Cell.

[b10] Urbich C, Kuehbacher A, Dimmeler S (2008). Role of microRNAs in vascular diseases, inflammation, and angiogenesis. Cardiovasc Res.

[b11] Thornton JE, Gregory RI (2012). How does Lin28 let-7 control development and disease?. Trends Cell Biol.

[b12] Sen CK, Gordillo GM, Khanna S (2009). Micromanaging vascular biology: tiny microRNAs play big band. J Vasc Res.

[b13] Zhu H, Shyh-Chang N, Segre AV (2011). The Lin28/let-7 axis regulates glucose metabolism. Cell.

[b14] Si R, Tao L, Zhang HF (2011). Survivin: a novel player in insulin cardioprotection against myocardial ischemia/reperfusion injury. J Mol Cell Cardiol.

[b15] Spence T, Perotti C, Sin-Chan P (2014). A novel C19MC amplified cell line links Lin28/let-7 to mTOR signaling in embryonal tumor with multilayered rosettes. Neuro Oncol.

[b16] Shinoda G, Shyh-Chang N, Soysa TY (2013). Fetal deficiency of lin28 programs life-long aberrations in growth and glucose metabolism. Stem Cells.

[b17] Ma H, Li SY, Xu P (2009). Advanced glycation endproduct (AGE) accumulation and AGE receptor (RAGE) up-regulation contribute to the onset of diabetic cardiomyopathy. J Cell Mol Med.

[b18] Sun D, Huang J, Zhang Z (2012). Luteolin limits infarct size and improves cardiac function after myocardium ischemia/reperfusion injury in diabetic rats. PLoS ONE.

[b19] Gao HK, Yin Z, Zhou N (2008). Glycogen synthase kinase 3 inhibition protects the heart from acute ischemia-reperfusion injury *via* inhibition of inflammation and apoptosis. J Cardiovasc Pharm.

[b20] Donahoe SM, Stewart GC, McCabe CH (2007). Diabetes and mortality following acute coronary syndromes. JAMA.

[b21] Miller BA, Hoffman NE, Merali S (2014). TRPM2 channels protect against cardiac ischemia-reperfusion injury: role of mitochondria. J Biol Chem.

[b22] Woodman OL, Long R, Pons S (2014). The cardioprotectant 3′,4′-dihydroxyflavonol inhibits opening of the mitochondrial permeability transition pore after myocardial ischemia and reperfusion in rats. Pharmacol Res.

[b23] Kristensen JM, Skov V, Petersson SJ (2014). A PGC-1α- and muscle fibre type-related decrease in markers of mitochondrial oxidative metabolism in skeletal muscle of humans with inherited insulin resistance. Diabetologia.

[b24] Henagan TM, Lenard NR, Gettys TW (2014). Dietary quercetin supplementation in mice increases skeletal muscle PGC1a expression, improves mitochondrial function and attenuates insulin resistance in a time-specific manner. PLoS ONE.

[b25] Fliss H, Gattinger D (1996). Apoptosis in ischemic and reperfused rat myocardium. Circ Res.

[b26] Fu J, Huang H, Liu J (2007). Tanshinone IIA protects cardiac myocytes against oxidative stress-triggered damage and apoptosis. Eur J Pharmacol.

[b27] Song JQ, Teng X, Cai Y (2009). Activation of Akt/GSK-3beta signaling pathway is involved in intermedin(1-53) protection against myocardial apoptosis induced by ischemia/reperfusion. Apoptosis.

[b28] Shyh-Chang N, Zhu H, Yvanka de Soysa T (2013). Lin28 enhances tissue repair by reprogramming cellular metabolism. Cell.

