# Supplementary material for: Lin28a protects against cardiac ischaemia/reperfusion injury in diabetic mice through the insulin-PI3K-mTOR pathway
Source: J Cell Mol Med. 2015 Feb 16;19(6):1174–82. doi: 10.1111/jcmm.12369 (PMC4459833; doi:10.1111/jcmm.12369)
Supplement: Supplementary file 1 [file jcmm0019-1174-sd1.docx]

**Supplementary Material**

Supplementary Table 1. Basic parameters of the mice

| Basic  parameters | Non-DM | | Sham | | I/R | | I/R+siControl | | I/R+siLin28a | | I/R+Control vector | | I/R+Lin28a | | I/R+Lin28a +RAP | |
| --- | --- | --- | --- | --- | --- | --- | --- | --- | --- | --- | --- | --- | --- | --- | --- | --- |
| Heart  rate(per min) | 335*.*8  (11*.*2) | 325*.*8  (15*.*0) | | 340*.*4  (13*.*1) | | 350*.*8  (10*.*0) | | 348*.*2  (9*.*2) | | 336*.*5  (12*.*5) | | 342*.*5  (11*.*5) | | 338*.*6  (10*.*2) | |  |
| Blood glucose(mmol/l) | 7*.*2  (1*.*3) | 17*.*8  (0*.*8) | | 18*.*2  (1*.*3) | | 18*.*5  （2*.*0） | | 17*.*5  （1*.*8） | | 18*.*2  （1*.*5） | | 18*.*4  （1*.*6） | | 17*.*8  （1*.*7） | |  |
| Body mass  (g) | 27*.*6  （1*.*5） | 35*.*9  （2*.*3） | | 34*.*7  （2*.*3） | | 34*.*3  （2*.*7） | | 35*.*3  （1*.*7） | | 34*.*1  （2*.*9） | | 36*.*3  （2*.*2） | | 35*.*8  （1*.*9） | |  |

Values are presented as mean（SD）.

Supplementary Table 2. CTs Values of Lin28a and Let7a expression

| CTs | Lin28a | GAPDH | Let7a | U6 |
| --- | --- | --- | --- | --- |
| Non-DM | 32.2(0.2) | 28.0(0.1) | 23.3(0.2) | 19.0(0.3) |
| DM | 36.9(0.3) | 32.2(0.2) | 25.0(0.3) | 21.2(0.4) |
| I/R | 37.9(0.2) | 32.4(0.1) | 24.3(0.3) | 21.0(0.4) |
| I/R+siControl | 35.5(0.4) | 30.0(0.3) | 24.7(0.2) | 21.3(0.3) |
| I/R+siLin28a | 36.8(0.4) | 30.1(0.3) | 25.9(0.1) | 23.0(0.2) |
| I/R+Control vector | 36.5(0.2) | 31.0(0.1) | 24.6(0.3) | 21.2(0.4) |
| I/R+Lin28a | 31.5(0.3) | 27.6(0.2) | 25.0(0.2) | 20.4(0.3) |
| I/R+Lin28a+RAP | 32.6(0.2) | 29.0(0.1) | 25.2(0.1) | 20.5(0.2) |

Values are presented as mean (SD).


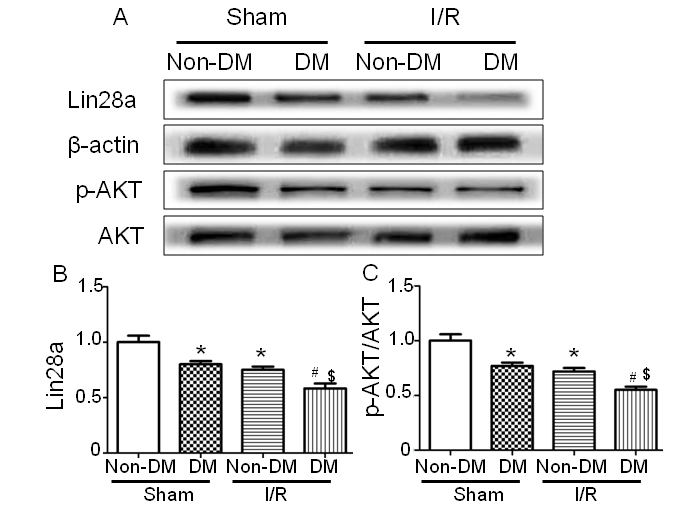


Supplementary Figure 1 Representative gel bolts depicting respective protein expression using specific antibodies (A); Lin28a (B); phosphorylated AKT (p-AKT) (C); The columns and error bars represent means and SD. n = 6 to 7 mice per group. *P <0.05 vs. Sham+Non-DM, ^#^P <0.05 vs. Sham+ DM. **^$^**P <0.05 vs. I/R + Non-DM.

^
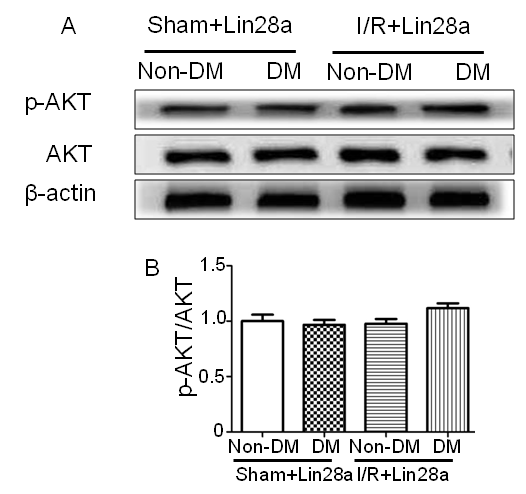
^

Supplementary Figure 2 Representative gel bolts depicting respective protein expression using specific antibodies (A); phosphorylated AKT (p-AKT) (B); The columns and error bars represent means and SD. n = 6 to 7 mice per group. No significant difference in p-AKT levels are found between groups.


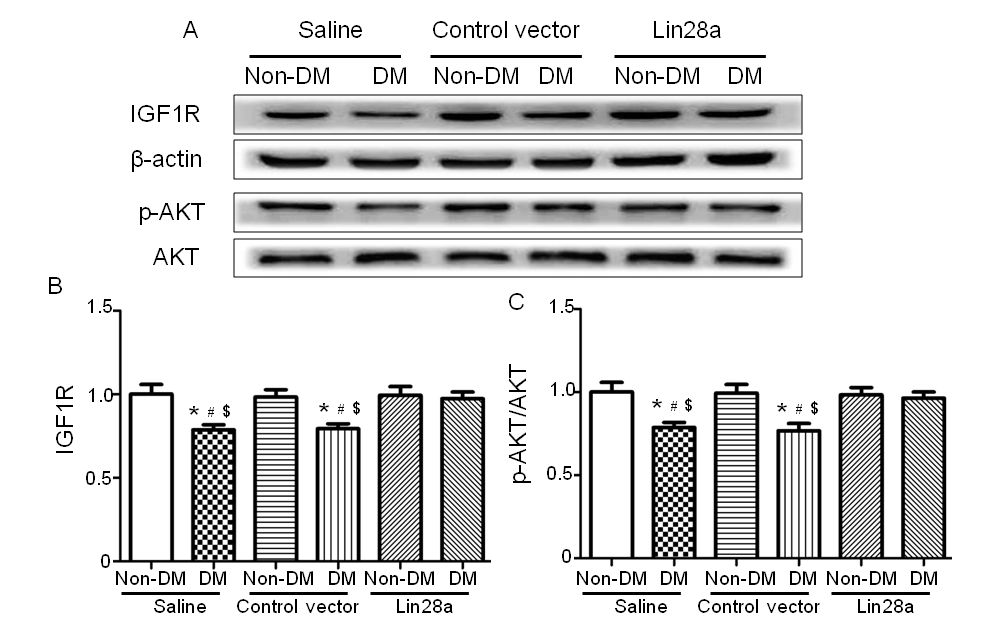


Supplementary Figure 3 Representative gel bolts depicting respective protein expression using specific antibodies (A); IGF1R (B); phosphorylated AKT (p-AKT) (C); The columns and error bars represent means and SD. n = 6 to 7 mice per group. *P <0.05 vs. Saline +Non-DM, ^#^P <0.05 vs. Control vector + Non-DM. **^$^**P <0.05 vs. Lin28a +DM.
